# Toxicity of POEA-containing glyphosate-based herbicides to amphibians is mainly due to the surfactant, not to the active ingredient

**DOI:** 10.1007/s10646-023-02626-x

**Published:** 2023-01-21

**Authors:** Zsanett Mikó, Attila Hettyey

**Affiliations:** Department of Evolutionary Ecology, Plant Protection Institute, Centre for Agricultural Research, Eötvös Loránd Research Network, Herman Ottó út 15, Budapest, 1022 Hungary

**Keywords:** Anura, Pesticide, Excipient, Life-history traits

## Abstract

Current international legislation regarding agrochemicals requires thorough toxicological testing mainly of the active ingredients. In a 96-h acute toxicity test we exposed *Rana dalmatina* and *Bufo bufo* tadpoles to either one of three concentrations of glyphosate, three concentrations of the surfactant (POEA), three concentrations of the two components together, or to non-contaminated water (control), and subsequently assessed mortality and body mass. To investigate whether simultaneous exposure to another stress factor influences effects of the contaminants, we performed tests both in the presence or absence of predator chemical cues. We found that the surfactant had significant harmful effects on tadpoles; survival was lowered by the highest concentration of the surfactant in case of *R. dalmatina*, while in *B. bufo* tadpoles it reduced survival already at medium concentrations. Body mass was significantly influenced by medium and high surfactant concentrations in both species. The presence of glyphosate did not have a significant effect by itself, but it slightly increased mortality in tadpoles exposed to medium concentrations of the surfactant in both species. The presence of chemical cues did not have an effect on the examined variables. Our study confirms that the toxicity of glyphosate-based herbicides is mainly due to the examined surfactant. Nonetheless, we found that glyphosate can enhance the harmful effect of the surfactant. These results stress that during the authorization process of new pesticide formulations, not only the active ingredients would need to be examined but the excipients should also be taken into account in an obligatory and systematic manner.

## Introduction

Millions of tons of pesticides are used worldwide every year, and considerable amounts reach non-agricultural habitats due to wind, wash-off by rain and inappropriate use (Pereira et al. 2009). Once they reach these areas, pesticides and their residues may harm non-target organisms by damaging their endocrine functions and immune system, exerting cytotoxic and teratogenic effects, leading to diminished reproductive success and survival (Giesy et al. 2000; Bianchi et al. 2006). Currently, in the European Union, the authorization of pesticides generally considers the possible negative effects of the active ingredients but not the formulations. However, excipients can also be toxic to non-target organisms and may have harmful effects on the environment. Despite their potential toxicity, excipients are usually regulated differently from active ingredients, furthermore ingredients inert in the main effect are generally not even indicated on product labels and are often claimed to be confidential business information (Klátyik et al. 2017). Although efforts have been made by regulatory authorities to progressively replace very toxic additives by less critical alternatives, these discrepancies in the pesticide authorization process can lead to serious problems because recommended application levels determined based solely on the toxicity of the active ingredients may often underestimate the toxic effects of commercial formulations.

Amphibians are considered an especially threatened vertebrate group (Stuart et al. 2004; Wake and Vredenburg 2008), with more than 40% of the species being at risk of extinction (IUCN 2021). The extensive use of pesticides has been proposed to be one of the major drivers of these declines (Davidson et al. 2002; Relyea 2005). It is their thin, highly permeable skin, unshelled eggs, and complex life-cycle that make amphibians especially vulnerable to pollutants both in the aquatic and the terrestrial environment. Also, amphibians use practically all types of water bodies for reproduction, which exposes eggs and larvae to the full spectrum of pesticides that reach natural surface waters. Nonetheless, amphibians have remained understudied with respect to environmental contaminants, since standard toxicity testing and authorization procedures require testing on fish but no other aquatic vertebrates (Adams and Rowland 2003; Nikinmaa 2014).

Glyphosate-based herbicides are among the most frequently applied pesticides worldwide (Relyea 2005; Grube et al. 2011), and, as a result of this, glyphosate is one of the three most often detected anthropogenic chemicals in freshwater ecosystems (Pérez et al. 2011). Previous studies showed that glyphosate-based herbicides are moderately to highly toxic to amphibians (Mann and Bidwell 1999). At sublethal concentrations they can slow development, hamper growth, and can also affect the behavior and body shape of animals, thereby leading to lowered fitness of individuals (Howe et al. 2004; Moore et al. 2012; Relyea 2012; Mikó et al. 2015).

Most glyphosate-based herbicide formulations recommended for terrestrial use contain some kind of surfactant. These compounds are primarily humectants, contributing to the homogeneous distribution of the herbicide on the surface of leaves, and facilitating the penetration of glyphosate through the cuticle layer (Bradberry et al. 2004). The most commonly used surfactants are polyethoxylated tallow amines (POEA). These tertiary amines contain two nitrogen atoms linked to polyoxyethylene (C_2_H_4_O) groups, and a long-chain alkyl group. The chain length and the saturation level of the alkyl group can vary, just like the chain length of the polyoxyethylene groups. Thus, POEA is not a homogeneous compound, but is a mixture of components having surfactant properties. The concentration of POEA in glyphosate-based herbicides varies between less than 1% and up to 21% (Bradberry et al. 2004).

Because glyphosate-based herbicides contain several ingredients, an important question related to their toxicity is whether it is caused by the active ingredient, glyphosate, or by the excipients, especially the surfactants. Previous studies suggested that for aquatic organisms POEA is the more harmful component (Folmar et al. 1979; Tsui and Chu 2003; Brausch and Smith 2007; Brausch et al. 2007; Frontera et al. 2011; Guilherme et al. 2012), but only a few studies involved amphibians and investigated the toxicity of both main components (Howe et al. 2004; Moore et al. 2012; Lanctôt et al. 2014). These studies compared the effects of the active ingredient and of the surfactant to that of the commercial formulations, which can contain several other components possibly exhibiting toxicity-modifying effects as well (Klátyik et al. 2017; Mesnage and Antoniou 2017). Furthermore, previous investigations did not assess the interactive effects of the components. Finally, all studies investigated toxic effects on anuran species of North America which may have undergone the most intense selection for tolerance to various compounds of the herbicide because of the widespread cultivation of genetically modified crops and the most extensive use of glyphosate-based herbicides, so that the generality of these observations remains unknown. Thus, there remains much to be learned about the magnitude of ecotoxicological effects of the two main components of glyphosate-based herbicides.

In this study we assessed the contribution of the main components of the most widely used formulations of glyphosate-based herbicides to their toxicity. To more closely model natural conditions, where usually several stress factors act in concert, we also examined how an additional stress factor (chemical cues on predation risk) may influence the toxicity of these components. Previous studies show that predation risk can increase mortality, reduce growth and development in the presence of pesticides (e.g. Relyea and Mills 2001; Relyea 2003a, 2004b, 2005). Hence, we exposed tadpoles of the agile frog (*Rana dalmatina*) and the common toad (*Bufo bufo*) to either one of three concentrations of glyphosate, three concentrations of POEA, three combinations of the two components, or to non-contaminated water, either in the presence or in the absence of chemical cues on predation risk. Both amphibian species are listed under the red list category “Least Concern” (IUCN), but they are protected in many European countries and may be considered useful model species in studies of amphibian ecotoxicology. The agile frog is widespread in Europe, it mainly inhabits deciduous woodlands, but also occurs in agricultural and urbanized areas. Nonetheless, many of its populations are currently in decline (Kaya et al. 2009). Common toads are also widespread in Europe and occupy a broad range of habitat types, including landscapes influenced by agriculture and urbanization. Although its populations are often large and stable, localized declines have been observed recently (Agasyan et al. 2009). We estimated treatment effects by assessing variation in body mass and mortality of tadpoles. We expected both components of glyphosate-based herbicides to be detrimental to tadpoles, with stronger malign effects of the surfactant (Howe et al. 2004; Moore et al. 2012; Lanctôt et al. 2014). We also predicted that the greatest effect would appear in the presence of both components, and that perceived predation threat would increase the negative effects of the components of the herbicide (Relyea 2005).

## Materials and methods

We collected 20 eggs from each of ten freshly laid egg-clutches of the agile frog (*Rana dalmatina*), and 10 days later 20 eggs from each of ten freshly laid egg-clutches of the common toad (*Bufo bufo*) from a pond in Nagykovácsi, Hungary (47° 34′ 35″ N, 18° 52′ 06″ E) and transported them to the Julianna-major Experimental Station (Plant Protection Institute, Centre for Agricultural Research, Eötvös Loránd Research Network) in Budapest (47°32′ 52″ N, 18°56′ 07″ E). Until hatching, we kept clutches one-by-one in the laboratory in 10-L containers holding 3 L of reconstituted soft water (RSW; USEPA 2002), at 20 °C and a 12:12 h light-dark cycle. We started the experiments when tadpoles reached the developmental stage 25 (Gosner 1960).

We captured eight dragonfly larvae (*Aeshna cyanea* Müller, 1764) from an artificial pond close to the experimental station (47° 33′ 04″ N, 18° 55′ 36″ E) and transported them to the laboratory. We kept dragonfly larvae individually in 300 mL cups holding 200 mL RSW and a wooden stick as a perching site. Predators were fed with bloodworms (*Chironomus* sp.) ad libitum every other day. Two days before the start of the experiment, we moved dragonfly larvae to 500 mL cups holding 400 mL RSW and from then onwards we fed them with two naive tadpoles every other day. On the first three occasions dragonflies received agile frog tadpoles, on the remaining occasions they were fed common toad tadpoles. We have successfully used very similar methods and concentrations in previous experiments (Mikó et al. 2015, 2017), and others were also able to induce phenotypic changes in tadpoles using these methods (Winkler and Van Buskirk 2012; Hanlon and Relyea 2013).

We started the first experiment by placing groups of 10 haphazardly selected, healthy-looking *R. dalmatina* tadpoles, one from each family, into 5-L containers filled with 4 L RSW. Temperature was set to 19 °C, lighting to a 13.5:10.5 h light-dark cycle. We exposed tadpoles to either 1, 2 or 4 mg acid equivalent (a.e.)/L glyphosate, to 0.44, 0.88 or 1.74 mL/L POEA, or to combinations of the two components (1 mg a.e./L glyphosate + 0.44 mL/L POEA, 2 mg a.e./L glyphosate + 0.44 mL/L POEA, or 4 mg a.e./L glyphosate + 1.74 mL/L POEA), and kept controls in non-contaminated RSW. The applied POEA concentrations reflected its proportion (in relation to the glyphosate content) in a popular formulation of a glyphosate-based herbicide (Glyphogan^®^ Classic; Monsanto Europe S.A., Brussels, Belgium) that contains 41.5 w/w% glyphosate and 15.5 w/w% POEA (Howe et al. 2004). According to ecotoxicological assessments, the observed worst-case concentration falls within the range between 1.7 and 5.2 mg a.e./L glyphosate in shallow surface water bodies, depending on habitat characteristics, and on the distance to agricultural lands (Giesy et al. 2000; Relyea 2012; Wagner et al. 2013). Based on the information available (Battaglin et al. 2005; Thompson et al. 2004), the applied herbicide concentrations represent pristine, intermediately and heavily contaminated habitats. Glyphosate and POEA were obtained from Sigma and LGC (analytical standards 337757 and DRE-E17136000), we prepared the stock solutions right before the start of the experiment by dissolving 40 mg glyphosate or 17.4 mL POEA in 10 mL deionized water. We exposed half of the individuals to chemical cues on predation risk by mixing the water from four predator cups at a time, and adding 20 mL from this mix to each container assigned to a treatment receiving chemical cues on predation risk. Resulting cue concentrations are known to be perceived by anuran tadpoles (Winkler and Van Buskirk 2012; Hanlon and Relyea 2013). In treatments receiving no cues, we added the same amount of RSW. We replicated each treatment six times in a randomized block design, resulting in a total of 120 experimental units. We did not change water during the course of the experiment, but fed tadpoles with chopped and slightly boiled spinach ad libitum. We visited each experimental unit daily, noted the number of live tadpoles and removed dead individuals. Four days after start we terminated the experiment, measured body mass of surviving tadpoles to the nearest 0.1 mg using a laboratory scale (Ohaus Pioneer PA114) and finally released animals at the site where egg-clutches had been collected from. Because animals were very small at the termination of the experiment (about 1 cm), we measured body mass of tadpoles in groups (survived individuals per container) to reduce measurement error. For the same reason, we did not measure body length of the animals. Changes in body length could have been an important result, but 96-h toxicity tests, which also assess developmental stage, usually use amphibian embryos, where development is much faster than in tadpoles (e.g., Sotomayor et al. 2012).

Two days after termination of the experiment on *R. dalmatina*, we repeated the experiment with *B. bufo* tadpoles applying the same treatments and methodology as described above (except that the light-dark cycle was set to 14:10 h to mimic natural light conditions).

### Statistical analyses

For the analysis of survival, we used Firth logistic regression, because data showed almost complete separation (fitted probabilities reached 0 or 1). We entered survival as the dependent variable, and treatment and block as fixed factors. To analyze variation in mean body mass we used linear mixed-effects models (LMM). We entered body mass as the dependent variable, treatment as a fixed factor, number of survived animals as a covariate, and block as a random factor. Because the design was not fully factorial (the concentrations of the components were not applied in all possible combinations) we performed planned comparisons using linear contrasts and controlled for repeated testing by correcting *P* values using the false discovery rate (FDR) method. We tested if the presence of chemical cues on predation risk can alter the effects of the components by comparing the predator vs. no predator treatments. Because this effect was not significant in either case (see Table 2), we disregarded the predator treatment and merged relevant replicates for the final analyses. We also compared the component-exposed treatments to the control group. Finally, to determine if the presence of glyphosate enhanced the toxicity of the surfactant, we compared the POEA-only and the glyphosate + POEA treatment groups. We also estimated LC_50_ values for both species separately for experiments and components (except for glyphosate, because mortality rate was very low) using generalized linear models (GZLM) with binomial error distribution and probit link function. Because in case of *R. dalmatina* the data showed almost complete separation (fitted probabilities reached 0 or 1), we used Bayesian generalized linear models (Gelman et al. 2008). We calculated 95% confidence intervals following Hackshaw (2009): exp (LC_50_ value ± 1.96 × standard error of the LC_50_ value). Statistical tests were performed with the “brglm” function of the “brglm” package and the “lme” function of the “nlme” package. In the post hoc analyses, we used the “emmeans” function of the “emmeans” package. To obtain LC_50_ values, we used the “glm” and “bayesglm” function of the “arm” package and “dose.p” function of the “MASS” package in “R”(version 3.6.1; R Core Team 2020).

## Results

**Fig. 1:**
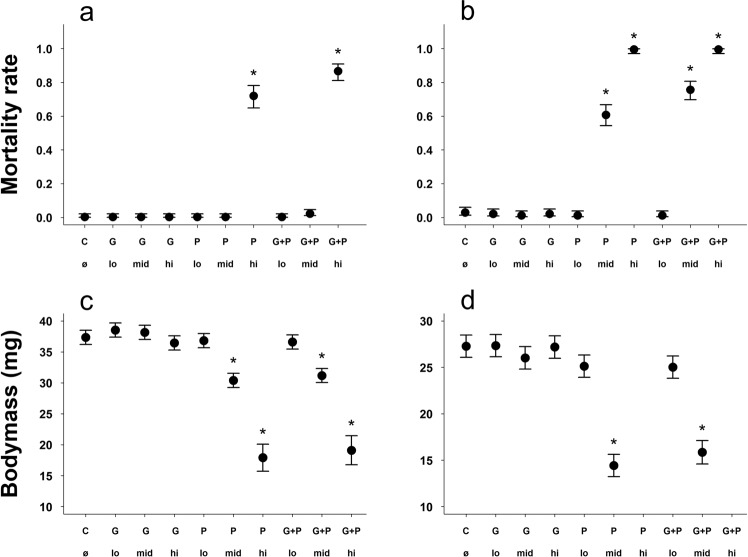
Survival and body mass of *R. dalmatina* (**a**, **c**) and *B. bufo* (**b**, **d**) tadpoles in the experimental treatment groups (C: control, G: glyphosate, P: POEA; lo: 1 mg a.e. glyphosate/L and/or 0.44 mL POEA/L, mid: 2 mg a.e. glyphosate/L and/or 0.88 mL POEA/L, hi: 4 mg a.e. glyphosate/L and/or 1.74 mL POEA/L). Error bars show the means and 84% CI estimated from linear mixed-effects models. Asterisks above error bars indicate the treatment groups significantly differing from the controls (*P* < 0.05)

In *R. dalmatina* tadpoles, survival was lowered by 74.2% in the treatment that contained the highest concentration of POEA, while we did not find significant effects of the other POEA concentrations or the treatments containing glyphosate only (Table 1 and Fig. 1). Survival of tadpoles exposed to the highest concentration of the combination of glyphosate and POEA was with 5% significantly lower than the survival of individuals exposed to the highest concentration of POEA only (linear contrasts, low concentration: *P* > 0.99, medium concentration: *P* = 0.19, high concentration: *P* = 0.04, Fig. 1). The presence of chemical cues on predation risk did not affect the lethality of either component (Table 2). The estimated LC_50_ values were 1.56 ± 0.028 mL POEA/L (mean ± SE, 95% CI: 1.48, 1.65) for the POEA-only treatment, and 1.39 ± 0.028 mL POEA/L (95% CI: 1.32, 1.48) for the glyphosate + POEA treatments.

**Table 1:** Summarizing table of the results of post hoc pairwise comparisons between the control and treatment groups on life-history traits of *R. dalmatina* and *B. bufo* tadpoles

	Survival				Body mass			
	ß	SE	*z*-ratio	*P*^a^	ß	SE	*z*-ratio	*P*^a^
*R. dalmatina*								
Control vs.								
Exposure to glyphosate								
Low concentration	<0.001	2.01	<0.001	>0.99	1.19	0.93	1.28	0.7
Medium concentration	<0.001	2.01	<0.001	>0.99	0.82	0.93	0.88	0.9
High concentration	<0.001	2.01	<0.001	>0.99	–0.89	0.93	–0.96	0.87
Exposure to POEA								
Low concentration	<0.001	2.01	<0.001	>0.99	–0.53	0.93	–0.57	0.98
Medium concentration	<0.001	2.01	<0.001	>0.99	–6.95	0.93	–7.47	<0.001*
High concentration	6.4	1.44	4.43	<0.001*	–19.45	1.66	–11.69	<0.001*
Exposure to glyphosate + POEA								
Low concentration	<0.001	2.01	<0.001	>0.99	–0.74	0.93	–0.79	0.93
Medium concentration	1.97	1.52	1.29	0.69	–6.17	0.93	–6.62	<0.001*
High concentration	7.02	1.44	4.87	<0.001*	–18.25	1.74	–10.49	<0.001*
*B. bufo*								
Control vs.								
Exposure to glyphosate								
Low concentration	–0.35	0.84	–0.41	0.99	0.06	1.03	0.06	>0.99
Medium concentration	–0.86	0.99	–0.87	0.9	–1.26	1.03	–1.22	0.68
High concentration	–0.35	0.84	–0.41	0.99	–0.09	1.03	–0.09	0.99
Exposure to POEA								
Low concentration	–0.86	0.99	–0.87	0.9	–2.17	1.03	–2.09	0.19
Medium concentration	3.95	0.58	6.86	<0.001*	–12.87	1.03	–12.47	<0.001*
High concentration	8.99	1.52	5.91	<0.001*	NA	NA	NA	NA
Exposure to glyphosate + POEA								
Low concentration	–0.86	0.99	–0.87	0.9	–2.27	1.03	–2.19	0.16
Medium concentration	4.65	0.59	7.95	<0.001*	–11.43	1.06	–10.83	<0.001*
High concentration	8.99	1.52	5.91	<0.001*	NA	NA	NA	NA

^a^*P* values are FDR corrected

*P < 0.05

**Table 2:** Summarizing table of the results of post hoc pairwise comparisons between the predator-free and predator-treatment groups on life-history traits of *R. dalmatina* and *B. bufo* tadpoles

	Survival				Body mass			
	ß	SE	*z*-ratio	*P*^a^	ß	SE	*t*-ratio	*P*^a^
*R. dalmatina*^b^								
C + no predator vs. C + predator	<0.001	1.97	<0.001	>0.99	–0.59	1.35	–0.44	0.66
GL + no predator vs. GL + predator	<0.001	1.97	<0.001	>0.99	0.53	1.35	0.39	0.69
GM + no predator vs. GM + predator	<0.001	1.97	<0.001	>0.99	–1.28	1.35	–0.95	0.35
GH + no predator vs. GH + predator	<0.001	1.97	<0.001	>0.99	–0.11	1.35	–0.08	0.94
PL + no predator vs. PL + predator	<0.001	1.97	<0.001	>0.99	2.27	1.35	1.67	0.09
PM + no predator vs. PM + predator	<0.001	1.97	<0.001	>0.99	–0.26	1.35	–0.19	0.85
PH + no predator vs. PH + predator	0.11	0.47	0.23	0.82	0.07	1.36	0.05	0.96
BL + no predator vs. BL + predator	<0.001	1.97	<0.001	>0.99	–0.19	1.35	–0.14	0.89
BM + no predator vs. BM + predator	1.99	1.49	1.33	0.18	–0.08	1.36	–0.06	0.96
BH + no predator vs. BH + predator	–0.16	0.57	–0.29	0.78	0.21	2.05	0.1	0.92
*B. bufo*^b^								
C + no predator vs. C + predator	–0.53	1.06	–0.49	0.61	0.03	1.51	0.02	0.99
GL + no predator vs. GL + predator	1.64	1.57	1.05	0.29	0.93	1.51	0.62	0.54
GM + no predator vs. GM + predator	1.12	1.66	0.67	0.5	0.64	1.51	0.42	0.67
GH + no predator vs. GH + predator	<0.001	1.18	<0.001	>0.99	–0.51	1.51	–0.34	0.73
PL + no predator vs. PL + predator	–1.12	1.66	–0.67	0.5	–0.95	1.51	–0.63	0.53
PM + no predator vs. PM + predator	–0.34	0.38	–0.92	0.36	–1.11	1.51	–0.74	0.46
PH + no predator vs. PH + predator	<0.001	2.03	<0.001	>0.99	NA	NA	NA	NA
BL + no predator vs. BL + predator	–1.12	1.66	–0.67	0.5	–0.03	1.51	–0.02	0.98
BM + no predator vs. BM + predator	–0.45	0.43	–1.04	0.29	–1.27	1.58	–0.8	0.43
BH + no predator vs. BH + predator	<0.001	2.03	<0.001	>0.99	NA	NA	NA	NA

^a^*P* values are FDR corrected

^b^C: control, GL: 1 mg a.e. glyphosate/L, GM: 2 mg a.e. glyphosate/L, GH: 4 mg a.e. glyphosate/L, PL: 0.44 mL POEA/L, PM: 0.88 mL POEA/L, PH: 1.74 mL POEA/L, BL: 1 mg glyphosate + 0.44 mL POEA, BM: 2 mg glyphosate + 0.88 mL POEA, BH: 4 mg glyphosate + 1.74 mL POEA

Mean body mass was also affected only in treatments where POEA was present: compared to controls we found a significant decrease by 18.2% in tadpole body mass at the medium concentration of POEA, while at the high POEA concentration, this decrease was 64.3% (Table 1 and Fig. 1). The additional presence of glyphosate did not significantly influence the effect of the surfactant on body mass of surviving tadpoles (linear contrasts, low concentration: *P* = 0.82, medium concentration: *P* = 0.4, high concentration: *P* = 0.27, Fig. 1). Chemical cues on predation risk did not influence the effect of the components on mean body mass (Table 2).

In *B. bufo* larvae, survival was decreased by 60.7% in the treatments containing the medium POEA concentration (Table 1), and in treatments containing the highest surfactant concentration, all tadpoles died before the end of the experiment (Fig. 1). Similar to *R. dalmatina* tadpoles, glyphosate increased the lethality of POEA at the medium concentration, namely to 75.6% (linear contrasts, low concentration: *P* > 0.99, medium concentration: *P* = 0.014, Fig. 1), and at the highest concentration the combined treatment also resulted in 100% mortality. The presence of chemical cues on predation risk did not affect significantly the effects of the components on mortality (Table 2). The estimated LC_50_ values were 0.82 ± 0.028 mL POEA/L (95% CI: 0.77, 0.87) in treatments containing only POEA, and 0.75 ± 0.029 mL POEA/L (95% CI: 0.71, 0.79) in the presence of both components.

Because all individuals died at the highest POEA concentration, we could not analyze its effect on body mass, but at the medium POEA concentration we found a significant decrease by 47.1% in the body mass of surviving tadpoles (Table 1). The presence of glyphosate did not change this effect significantly (linear contrasts, low concentration: *P* = 0.92, medium concentration: *P* = 0.18, Fig. 1). The presence of chemical cues on predation risk also did not have a significant effect (Table 2).

## Discussion

Our results clearly show that from the two main components of the most widely used glyphosate-based herbicides, it is the surfactant (POEA) that is primarily responsible for the harmful effects, while the active ingredient (glyphosate) has much weaker toxicity. POEA can cause haemolysis, lipid peroxidation and it can damage the DNA (Navarro and Martinez 2014; de Brito Rodrigues et al. 2019). Due to its surfactant property, POEA can increase membrane permeability (Hedberg and Wallin 2010) and decrease absorption of glycerol and fatty acids in the intestine (Frontera et al. 2011). We found significant effects only in treatment groups where the surfactant was present; survival was affected only by the highest concentration of POEA in case of *R. dalmatina*, while in *B. bufo* tadpoles, it reduced survival already at medium concentrations. Body mass was significantly influenced by both medium and high surfactant concentrations in both species. The presence of glyphosate slightly increased the lethality of the surfactant in both species, but a similar effect on body mass of surviving tadpoles was not detectable. The presence of chemical cues on predation risk did not alter effects of either component in itself or in combination.

A good number of studies investigated effects of the components of glyphosate-based herbicides on amphibians, but in several of these experiments only the toxicity of glyphosate per se (Mann and Bidwell 1999; Hedberg and Wallin 2010; Rissoli et al. 2016) or the surfactant in itself (Perkins et al. 2000; Edginton et al. 2004a) was compared to the commercial formulations. Other studies compared toxic effects of various formulations containing different types of surfactants (Fuentes et al. 2011; Lajmanovich et al. 2011; Edge et al. 2014). Studies allowing for a direct comparison of the toxicity of the two most common components of glyphosate-based herbicides, glyphosate and POEA have remained scarce. Howe et al. (2004) examined the effects of five different formulations of glyphosate-based herbicides, as well as that of glyphosate and of POEA in themselves on *Rana clamitans* tadpoles. They found that treatments containing POEA caused the highest mortality, while herbicide formulations not containing this surfactant, as well as glyphosate per se became lethal only at very high concentrations (above 17.5 mg a.e./L). Moore et al. (2012) exposed tadpoles of five North-American frog species (*R. catesbeiana*, *R. clamitans*, *R. pipiens*, *Anaxyrus fowleri* and *Hyla chrysoscelis*) to a glyphosate-based herbicide and its two main components, glyphosate and POEA. They could attribute practically 100% of the toxicity of the herbicide to the surfactant. Lanctôt et al. (2014) examined *Rana sylvatica* tadpoles at two developmental stages (25 and ~30) and exposed them either to one of two glyphosate-based herbicides, to glyphosate in the form of isopropylamine (IPA) salt alone, or to POEA alone throughout the whole experiment. Similar to previous studies, they observed the highest mortality in the chronic presence of the surfactant. However, neither of these studies examined the interactive effect of glyphosate and POEA. Our results are consistent with those of previous studies in that the toxic effects of glyphosate-based herbicides were largely attributable to POEA. Furthermore, by investigating the interactive effects between the main ingredient and the surfactant, we also showed that glyphosate, when applied in combination with POEA (as is the case in many glyphosate-based herbicides), can further increase POEA-caused lethality. It is assumed that POEA acts synergistically with glyphosate at the mitochondrial level (Peixoto 2005). Frontera et al. (2011) indeed observed a larger decrease in oxygen consumption of *Cherax quadricarinatus* exposed to mixtures of POEA and glyphosate, which they attributed to enzyme inhibition, and which resulted in lowered protein levels and decreased somatic growth (Frontera et al. 2011).

Beside the effect on survival, treatments containing medium and high concentrations of POEA also had sublethal effects: they decreased the body mass of surviving tadpoles in both species. This negative effect on body mass has also been observed in former studies that investigated the effects of POEA-containing glyphosate-based herbicides (Relyea 2004a; Cauble and Wagner 2005; Mikó et al. 2015) and may partly be attributed to toxicity (see above), costs of detoxification and to lowered activity resulting in decreased food intake (Moore et al. 2015; Mikó et al. 2017). However, from among the studies that investigated the toxicity of the surfactant separately, only one examined its effect on the mass of animals (Lanctôt et al. 2014): they observed a slight increase in body mass in young tadpoles (developmental stage 31), but not in older larvae. In contrary, we observed a negative effect of POEA on tadpole mass. The apparent positive effect of the herbicide as observed by Lanctôt et al. (2014) may have arisen because tadpoles were kept in groups: increased mortality causing lowered frequencies of direct interactions and decreased competition for food have resulted in the reported increase in body mass (Alford 2000). Furthermore, because glyphosate-based pesticides can decrease tadpole activity (Moore et al. 2015; Mikó et al. 2017), it is also possible that the temporary increase in tadpole mass was due to enhanced feeding after individuals were released from the suppressive effect of the surfactant (as their last exposure took place when tadpoles were at ca. developmental stage 30).

While earlier studies have shown that the harmful effects of glyphosate-based herbicides may be influenced decisively by both biotic and abiotic factors (Sparling 2003; Chen et al. 2004; Edginton et al. 2004b; Wojtaszek et al. 2004; Jones et al. 2011; Mikó et al. 2015), results of experiments that contained exposure to predation threat as an extra stress factor remained contradictory (Relyea 2005, 2012). In our experiment, chemical cues indicating predation threat did not affect the toxicity of the herbicide components. While the applied cue concentrations (18.75 mg tadpoles L^–1^ week^–1^) are known to be perceived very well by tadpoles and to elicit clear antipredator responses (Winkler and Van Buskirk 2012; Hanlon and Relyea 2013), it is possible that the effect of predation threat on mortality and body mass was not detectable because of the brevity of exposure: previous experiments delivering a significant effect lasted for at least 16 days (Relyea and Mills 2001; Relyea 2003a, 2004b, 2005). Although tadpoles respond instantly to the appearance of chemical cues of predation threat by altering their behavior (Relyea 2003b; Orizaola et al. 2012; Van Buskirk et al. 2014), the development of morphological responses is a slower process (Van Buskirk and Arioli 2002; Relyea 2003b; Orizaola et al. 2012). Therefore, further investigations are required to decide to what extent and under what circumstances predation threat may intensify malign effects of herbicides.

In summary, our study confirms that in POEA-containing herbicides the surfactant is much more toxic to amphibian larvae than the active ingredient. Our study, however, also delivers evidence that glyphosate can enhance the malign effect of the surfactant. Finally, we did not find evidence for predation threat enhancing the toxicity of the active ingredient, of the surfactant, or their combination. Consequently, the toxicity of glyphosate-based herbicides is likely to depend mainly on the amount of the surfactant present in the formulation. However, surfactant types other than POEA that are present in marketed formulations or are to become ingredients of new formulations in the future may be even more harmful to amphibians (Perkins et al. 2000; Howe et al. 2004; Fuentes et al. 2011; Lanctôt et al. 2014). Unfortunately, producers of pesticides are not always indicating the exact composition of their products in all countries. This significantly increases the uncertainty about the proper use of herbicides and enhances the chance and potential severity of environmental destruction. Consequently, studies assessing environmental impacts of the marketed herbicide formulations and their ingredients will remain in need. Even more importantly, during the authorization process of new pesticide formulations, not only the active ingredients would need to be systematically tested, but the toxicity of the excipients should be taken into account to a large extent.
